# Effect of 0.01% atropine combined with orthokeratology lens on axial elongation: a 2-year randomized, double-masked, placebo-controlled, cross-over trial

**DOI:** 10.3389/fmed.2024.1358046

**Published:** 2024-04-23

**Authors:** Binbin Li, Shiao Yu, Shasha Gao, Guangli Sun, Xuena Pang, Xiuhong Li, Ming Wang, Fengyan Zhang, Aicun Fu

**Affiliations:** ^1^The First Affiliated Hospital of Zhengzhou University, Zhengzhou, China; ^2^The Affiliated Eye Hospital of Suzhou Vocational Health College, Suzhou, China; ^3^Beijing Aier Intech Eye Hospital, Beijing, China

**Keywords:** myopia, orthokeratology, 0.01% atropine, axial length, schoolchildren

## Abstract

**Purpose:**

To evaluate the effect of 0.01% atropine combined with orthokeratology (OK) lens on axial elongation in schoolchildren with myopia.

**Methods:**

Sixty children aged 8–12 years with spherical equivalent refraction (SER) from -1.00D to -4.00D in both eyes were enrolled in this randomized, double-masked, placebo-controlled, cross-over trial. Children who had been wearing OK lenses for 2 months were randomly assigned into combination group (combination of OK lens and 0.01% atropine) for 1 year followed by control group (combination of OK lens and placebo) for another 1 year or vice versa. This trial was registered in the Chinese Clinical Trial Registry (Number: ChiCTR2000033904, 16/06/2020). The primary outcome was changes in axial length (AL). Data of right eyes were analyzed.

**Results:**

There were statistically significant differences in the changes in AL between combination and control groups after generalized estimating equation model adjusting for age and baseline SER (*p* = 0.001). The mean axial elongation difference between combination and control groups was 0.10 mm in the first year (0.10 ± 0.13 mm vs. 0.20 ±0.15 mm; *p* = 0.01), and 0.09 mm in the second year (0.22 ± 0.10 mm vs. 0.13 ± 0.14 mm; *p* = 0.01), respectively. The mean axial elongation difference of two groups in the first year was similar to that in the second year during the cross-over treatment.

**Conclusion:**

In central Mainland China in myopic children, the treatment of combination therapy is more effective than single OK lens in controlling axial elongation.

## 1 Introduction

The prevalence of myopia has reached alarming levels (1–3). Rapid myopia progression is associated with a significantly increased risk of myopia-related complications (4–8). Studies have found that low concentrations of atropine and orthokeratology (OK) lenses may delay axial elongation and myopia progression (9, 10). Furthermore, studies including the pre-cross-over period of the current study have reported that OK lenses combined with low concentrations of atropine are more effective than using OK lenses alone in controlling axial elongation (11–13). In this study, a cross-over design further confirmed the efficacy of combination therapy. There has been a wide use of cross-over design in clinical trials, including studies of myopia control (14, 15). With this method, participants serve as controls, statistically eliminating between-subject variability in myopia control trials based on genetic and environmental factors, providing greater statistical power (or requiring a smaller sample size) than a parallel-group design (16). Using a cross-over design, this study compares the efficacy of combined treatment with single OK lens treatment, providing scientific evidence for the clinical application of combined treatment.

## 2 Methods

### 2.1 Study design

This randomized, double-masked, placebo-controlled, cross-over trial comprised two periods. The detailed study protocol and methods have been described previously in period 1 (11). Sixty Chinese myopic children (Han nationality) who visited the First Affiliated Hospital of Zhengzhou University between June 2020 and September 2020 were recruited for this trial. In brief, aged 8–12 years, spherical equivalent refraction (SER) from −1.00D to −4.00D in both eyes, astigmatism no more than 1.50D, anisometropia of ≤1.00D, monocular best corrected visual acuity of ≥20/20 (Snellen chart), and intraocular pressures (IOPs) < 21 mmHg (17–27). In addition, there were no other eye diseases or surgery, and no eye or systemic organic changes affecting vision acuity were enrolled. This trial was approved by the Human Ethics Committee of the First Affiliated Hospital of Zhengzhou University (Number: 2020-KY-223), registered in the Chinese Clinical Trial Registry (Number: ChiCTR2000033904, 16/06/2020), and adhered to the tenets of Declaration of Helsinki.

Participants who had been wearing OK lenses successfully for 2 months (as baseline) were randomly assigned in a 1:1 ratio to the combination group (combination of OK lenses and 0.01% atropine eye drops) for 1 year in period 1 followed by the control group (combination of OK lenses and placebo) for another 1 year in period 2 or vice versa. Details of the preparation method of 0.01% atropine and placebo eye drops have been published elsewhere and are available in the Supplementary File (28, 29). OK lenses were four-zoned reverse-geometry lenses (Boston EM Material, Alpha Corp, Nagoya, Japan) with a 6.0 mm light area diameter. OK lenses were routinely replaced appropriately every 1.5 years, but the lenses would be reordered when the residual SER (sphere plus half of the cylindrical power) exceeded 0.50 D at any visit.

Stratified block randomization was used to control for SER and age at enrollment. A total of 0.01% atropine and placebo eye drops were packaged in identical bottles, maintained, and distributed by the same doctor. To reduce observation bias, the data analysts were blinded.

### 2.2 Study procedures

In period 2, children underwent the same standardized examinations as in period 1. Axial length (AL), corneal curvature, corneal astigmatism, and anterior chamber depth (ACD) were obtained using a non-contact partial coherence interferometer (IOL-Master 500: 7.7.4 software version, 1.3375 Group Refractive Index, Carl Zeiss Meditec AG, Germany). The signal-to-noise ratio for AL readings is greater than 2.0. Pupil diameter was measured using an autorefractor (NIDEK, AR-1, Japan). Accommodative amplitude was measured monocularly using the push-up technique. IOP was measured using a non-contact tonometer (TX-20, 1.5.1.0 software version, Canon, Japan). Details of the examination methods for cycloplegic autorefraction, AL, corneal curvature, corneal astigmatism, ACD, pupil diameter, accommodative amplitude, IOP, and discomfort symptoms have been published elsewhere and are available in the Supplementary File (28, 29). The primary outcome was changes in AL.

At the randomized visit and subsequent 4-month follow-up, each participant was given four bottles of eyedrops. OK lenses, lens suction holders, lens cases, and care solutions were checked and recorded at each visit. The average weekly use of eye drops and the wearing of OK lenses were assessed using a paper questionnaire.

### 2.3 Statistical analyses

The sample size was calculated based on the results of previous studies (12, 13, 30). We assumed that 90% power was required to detect at least 0.10 mm AL difference between the combination and control groups, with significance at the two-sided 5% level and standard deviation of 0.15 mm, a sample size of 48 participants is needed. By factoring in an attrition rate of 20%, a sample size of 60 participants is needed.

All statistical analyses were performed using SPSS software (version 26.0, Chicago, Illinois, United States) and R software (version 4.3.2, R Foundation for Statistical Computing, Beijing, China). The right eye data were analyzed based on the intention-to-treat principle. The baseline data were tested for normality using the Kolmogorov–Smirnov test. An independent t-test was used to compare the change in AL between the combination and control groups in periods 1 and 2. A generalized estimating equation model was used to compare the efficacy of the two groups. A *p*-value of <0.05 was considered statistically significant.

## 3 Results

**Figure 1:**
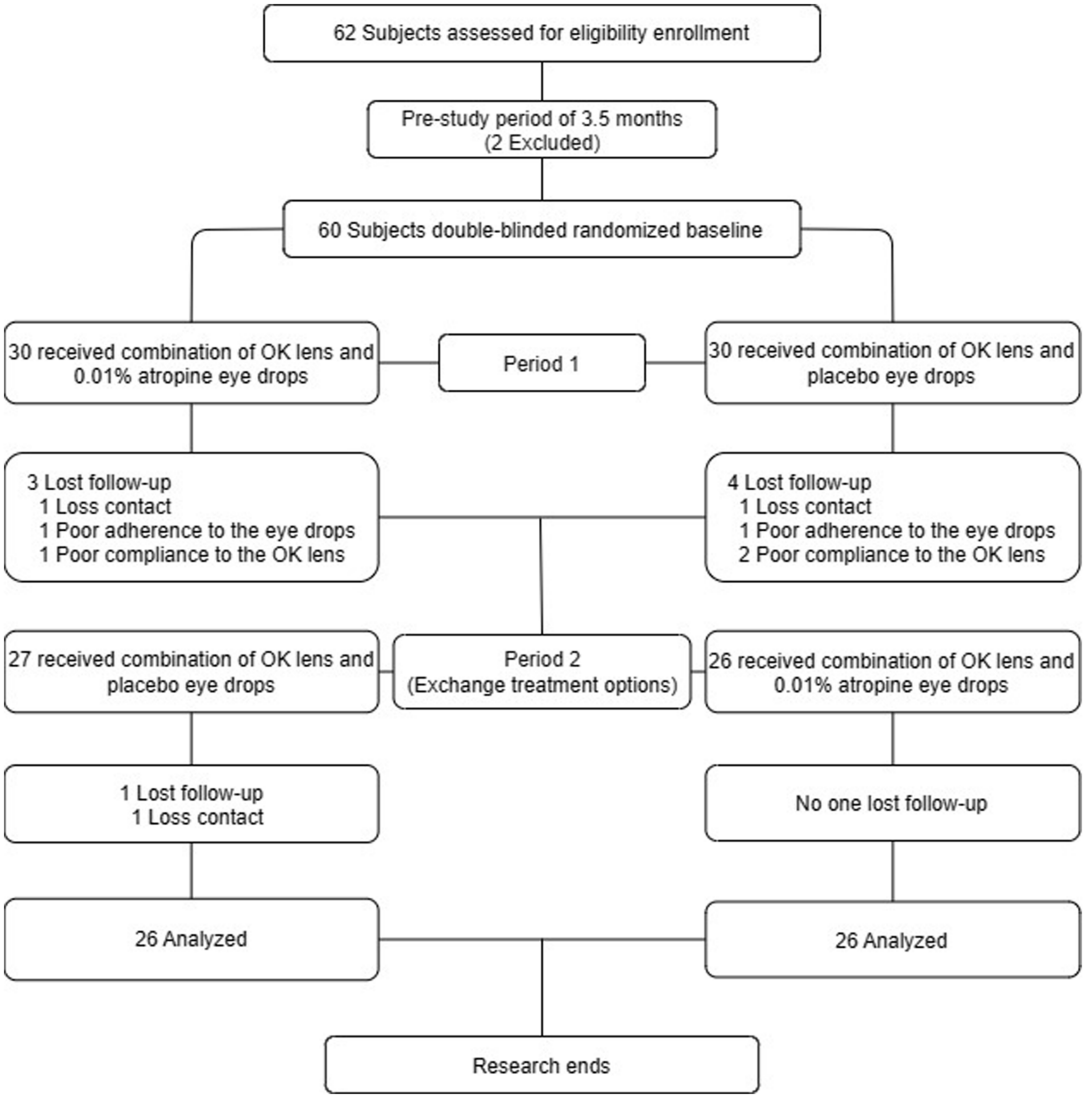
Participant recruitment and randomization flowchart.

Sixty participants enrolled in this trial. Figure 1 shows the randomization of the individuals and study outline for periods 1 and 2. There were no significant differences in baseline characteristics between the combination and control groups (Table 1). Fifty-three and 52 participants successfully completed period 1 and period 2 visits, respectively. Seven participants in period 1 (three in the combination group and four in the control group) and one participant (control group) in period 2 dropped out.

**Table 1:** Characteristics at enrollment of participants in two groups followed for 2 years, mean ± SD or n (%).

Variables	Combination first group (*n* = 26)	Control first group (*n* = 26)	*p*
Age (year)	9.95 ± 1.49	9.80 ± 1.64	0.73
Spherical equivalent refraction (SER, D)	−2.77 ± 0.89	−2.81 ± 0.97	0.88
Body mass index (kg/m^2^)	18.77 ± 4.03	17.48 ± 3.40	0.22
Age at myopia diagnosis (year)	8.71 ± 1.56	8.84 ± 1.66	0.77
Age at first wearing of glasses (year)	8.94 ± 1.54	8.83 ± 1.59	0.80
SER progression 1 year before study enrollment (D)	−0.79 ± 0.44	−0.77 ± 0.38	0.86
Corneal curvature (D)	42.74 ± 1.31	42.88 ± 1.25	0.69
Corneal astigmatism (D)	1.14 ± 0.51	1.23 ± 0.44	0.51
Anterior chamber depth (ACD, mm)	3.62 ± 0.45	3.70 ± 0.25	0.41
Intraocular pressure (IOP, mmHg)	16.79 ± 3.05	16.47 ± 2.43	0.19
Pupil diameter (mm)	5.63 ± 1.41	5.72 ± 1.19	0.80
Accommodative amplitude (D)	16.02 ± 3.85	15.99 ± 4.03	0.98
Axial length (mm)	24.71 ± 0.75	24.64 ± 0.79	0.73
Male, n (%)	10 (38.46%)	15 (57.69%)	0.17
Heredity			0.53
- - (neither parent myopic)	2 (7.69%)	5 (19.23%)	
+ − (one parent myopic)	11 (42.31%)	9 (34.62%)	
+ + (both parents myopic)	13 (50.00%)	12 (46.15%)	

### 3.1 Changes in AL in periods 1 and 2

**Figure 2:**
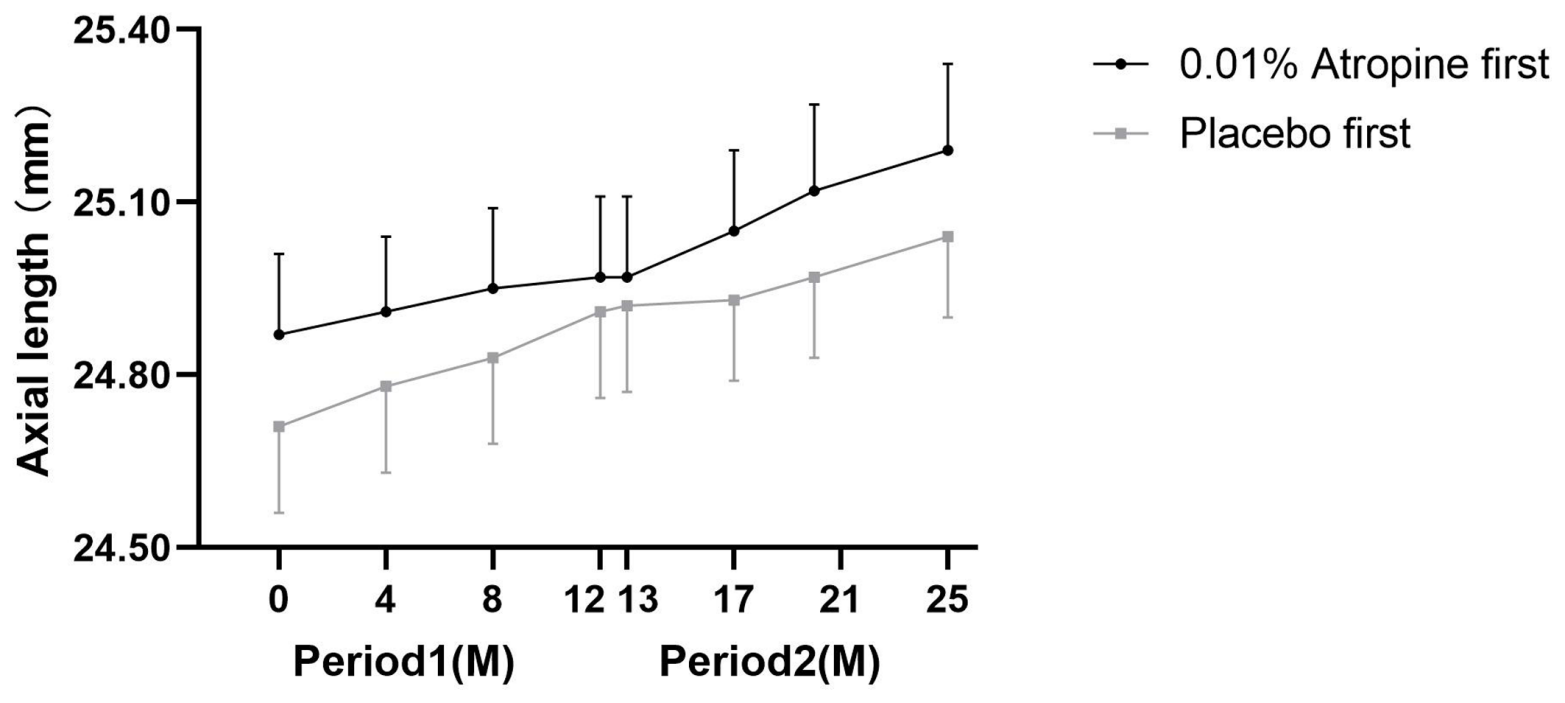
Axial length at each follow-up time point in both groups. M = month.

There were statistically significant differences in the changes in AL between combination and control groups after the generalized estimating equation model adjusting for age and baseline SER (*p* = 0.04), with a mean AL difference of 0.10 mm (0.10 ± 0.13 mm vs. 0.20 ± 0.15 mm; *p* = 0.01) in period 1, and 0.09 mm (0.22 ± 0.10 mm vs. 0.13 ± 0.14 mm; *p* = 0.01) in period 2 (Table 2). Figure 2 shows the AL of the participants at each time point. The mean changes in AL were 0.04 ± 0.06 mm, 0.08 ± 0.11 mm, and 0.10 ± 0.13 mm in the combination group and 0.07 ± 0.08 mm, 0.13 ± 0.11 mm, and 0.20 ± 0.15 mm in the control group during 4, 8, and 12 months in the first year, respectively. The corresponding changes were 0.03 ± 0.04 mm, 0.07 ± 0.10 mm, and 0.13 ± 0.14 mm in the combination group, and 0.08 ± 0.06 mm, 0.14 ± 0.05 mm, and 0.22 ± 0.10 in the control group during 4, 8, and 12 months in the second year. The mean AL difference between the combination and control groups in period 1 was similar to that in period 2 during the cross-over treatment. Over 2 years, the mean axial elongation was 0.33 ± 0.19 mm and 0.33 ± 0.25 mm in the combination first and control first groups, respectively (*p* = 0.90).

**Table 2:** Changes in axial length in two groups at each period (mean ± SD).

Study period	Combine first group (*n* = 26)	Control first group (*n* = 26)	*p*
Period 1	0.10 ± 0.13	0.20 ± 0.15	0.04
Period 2	0.22 ± 0.10	0.13 ± 0.14	

### 3.2 Adverse events

No serious adverse events were found in period 2, as reported in period 1 by us previously. Four children complained of mild photophobia in bright sunlight within 1 month after switching to the combined group from the control group. Three and two children in the switch to control and combined groups had moderate superficial punctate keratopathy, but these conditions completely recovered after withdrawal of OK lens wear for 1 week.

## 4 Discussion

To our knowledge, this is the first double-masked cross-over study aimed at exploring the comparative efficacy of combination therapy and single OK lens therapy. This clinical study demonstrated that combination therapy is more effective than a single OK lens in controlling axial elongation.

Previous studies with different designs from the current study have reported the add-on effect of OK lenses combined to 0.01% (12, 13, 30). Data before cross-over (period 1) from the present trial showed that combination therapy was more effective than OK lenses alone in preventing axial elongation (11). The changes in AL in the combination group were lower than 0.10 mm in period 1 and 0.09 mm in period 2 compared to the control group. Kinoshita et al. (13) randomized 80 children with a SER of -1D to -6D into the combination group (OK and 0.01% atropine ophthalmic solution) and the control group (OK monotherapy). The 2-year AL progression was found to be 0.29 ± 0.20 mm and 0.40 ± 0.23 mm in the combination and control groups, respectively. In the present study, the AL progression was 0.10 ± 0.13 mm and 0.13 ± 0.14 mm in the combination group and control group in period 1 and period 2, respectively, and 0.20 ± 0.15 mm and 0.22 ± 0.10 mm in the control group, respectively, which was similar to the results of the present study.

The interaction between intra-group comparisons when children switched between combination and control groups, and inter-group comparisons before and after cross-over was not statistically significant in the current study, suggesting that the treatment effect of combination therapy did not change over time. In other words, the combination treatment has better effects than OK lenses alone, both in periods 1 and 2. Thus, clinically, for fast axial elongation and poor responders of OK lenses alone, combining 0.01% atropine may be considered to control axial elongation. The mechanism by which OK lenses combined with 0.01% atropine are more effective than OK lenses alone is unclear. This may be a consequence of the combined action of both pharmacological and optical mechanisms, where atropine-induced pupil dilation can increase myopic defocus induced by OK lenses (31–34). Additionally, studies have found that 0.01% atropine eye drops and OK lenses can increase choroidal thickness, and the combination of OK lenses and 0.01% atropine induced a greater increase in choroidal thickness than monotherapy with 0.01% atropine or OK lenses, and the more choroidal thickening was associated with the slower SER progression and axial elongation in the combination group (26, 35–37). Hao et al. (26) found that the subfoveal choroidal thickness (SF-ChT) increased by 5.41 μm, 17.46 μm, and 20.19 μm after 1 month, and AL increased by 0.20 mm, 0.28 mm, and 0.14 mm after 12 months in the 0.01% atropine, OK lenses, and combination of 0.01% atropine and OK lens groups, respectively, and the changes in SF-ChT at 1 month were negatively correlated with the changes in AL at 12 months. Zhao et al. (37) observed that the SF-ChT increased more in the combination of OK and 0.01% atropine and OK lens alone groups, followed by the 0.01% atropine group while decreasing in the control group after 1-month follow-up.

In the current study, refraction was not measured in two groups after steps 1 and 2. This decision stemmed from the necessity for children to discontinue wearing OK lenses for a duration exceeding 1 month (38–40), allowing their corneas to revert to their original shape, and thus ensuring relatively accurate refraction assessment. Ceasing OK lens wear for just one night can precipitate a decline in daytime visual acuity, while abstaining from wearing OK lenses for over a month may induce shifts in SER and AL. Consequently, neither the children nor their guardians were inclined to subject them to such a prolonged cessation of OK lens wear. Moreover, similar to the current study, most investigations on the efficacy of OK lenses in halting myopia progression in children predominantly relied on AL changes as a surrogate measure of efficacy, while refraction change over time was not systematically observed (13, 41, 42).

The strengths of the current study were the randomized, double-masked, cross-over nature of the trial, in which all participants had the opportunity to use combination therapy, which therefore enabled comparisons both within and across the groups. However, this study has some limitations. First, although randomized and designed for cross-over after 1-year combined use of 0.01% atropine, the short duration of the trial does not permit evaluation of the long-term performance of the combination therapy. Second, 0.05 and 0.02% atropine were more effective than 0.01% atropine (28, 43), but they were all well tolerated without an adverse effect. Reduced treatment zone OK lenses are more effective in delaying axial elongation compared with conventional OK lenses (44). Third, the rebound effect of 0.01% atropine or OK lenses could not be observed in this cross-over design study (45, 46). Therefore, further studies including higher atropine concentrations combined with different treatment zones of OK lenses should be conducted to observe the effect and rebound effect of withdrawal atropine in the combination therapy. Additionally, studies have shown that the growth of AL in children gradually slows down with age, which may have an impact on the results of this study, so it is necessary to increase the sample size and stratify the age for further comparison in the future to validate the results of this study (47). Finally, the IOL-Master device uses a single mean group refractive index (GPI) for the entire eye, instead of calculating the segmented AL as the sum of geometrical ocular segments converted from the optical path length in each medium. The AL results (with longer values) will be inaccurate in long eyes with an AL of 26 mm or more when using GRI-based measurements (48–50). However, the children we recruited did not have high myopia (SER from −1.00D to −4.00D), and there was only one child at baseline (26.12 mm) and two children at 2 years after treatment (26.31 and 26.44 mm) with AL greater than 26.0 mm in the current study. The results of this study did not change if we removed these two children for re-analysis.

In conclusion, during the 2-year cross-over trial in central China, the treatment of combination therapy was more effective than a single OK lens in controlling axial elongation.

## Data availability statement

The original contributions presented in the study are included in the article/Supplementary material, further inquiries can be directed to the corresponding authors.

## Ethics statement

The studies involving humans were approved by Human Ethics Committee of the First Affiliated Hospital of Zhengzhou University (Number: 2020-KY-223). The studies were conducted in accordance with the local legislation and institutional requirements. Written informed consent for participation in this study was provided by the participants’ legal guardians/next of kin. Written informed consent was obtained from the minor(s)’ legal guardian/next of kin for the publication of any potentially identifiable images or data included in this article.

## Author contributions

BL: Writing – original draft, Writing – review & editing, Data curation. SY: Data curation, Writing – review & editing. SG: Writing – review & editing. GS: Writing – review & editing. XP: Writing – review & editing. XL: Writing – review & editing. MW: Writing – review & editing. FZ: Writing – review & editing. AF: Data curation, Funding acquisition, Writing – original draft, Writing – review & editing.
