# Supplementary material for: Effect of 0.01% atropine combined with orthokeratology lens on axial elongation: a 2-year randomized, double-masked, placebo-controlled, cross-over trial
Source: Front Med (Lausanne). 2024 Apr 23;11:1358046. doi: 10.3389/fmed.2024.1358046 (PMC11074463; doi:10.3389/fmed.2024.1358046)
Supplement: Supplementary file 2 [file Data_Sheet_2.PDF]

The details of the examination method for the observation items (AL, SER, accommodation amplitude and pupil diameter) and questionnaire of discomfort symptoms

The AL, corneal power, and anterior chamber depth were evaluated using a non-contact partial coherence interferometer (IOL-Master; Carl Zeiss Meditec AG, Germany). Five successive measurements were taken on each occasion, and their means were used for analysis. Accommodation amplitude was measured monocularly by the push-up technique. Children wore their fully corrected spectacle prescription where prescribed and focused on the previous line of best-corrected visual acuity in the right eye while the left eye was occluded. Children were instructed to focus on the letter as the chart moved closer. They were told to keep the letter as clear as possible until they could no longer be held with clear focus. The inverse of the final distance in meters was recorded as the accommodation amplitude of the child. The accommodation amplitude was recorded three times, and the average was noted. Pupil diameter was measured using an autorefractor (NIDEK, AR-1, Japan) under bright light indoors. The light in the examination room was maintained at constant illumination of 300–310 lux (TES-1332A illumination photometer). Children had to adapt to ambient light in the examination room for 10 min before the measurement. Three consecutive measurements were performed, and the average values were recorded. IOP was measured using a non-contact tonometer (TX-10, Canon, Japan) as the mean of five measurements. Cycloplegic autorefraction was performed using four drops of compound tropicamide eye drops (0.5% tropicamide and 0.5% neo-synephrine) (Santen, Japan) administered at 10-minute intervals in each of the participants' eyes. Ten minutes after the last drop, cycloplegic autorefraction was measured three times by an

autorefractor (NIDEK, AR-1, Japan). Three readings, all within a difference of 0.25 D, were averaged for analysis.

How often are you experiencing aversion to light? (never, occasionally, often, always); how severe is the aversion to light? (zero, normal indoor, daily outdoor, bright sun light); (2) How often do you experience blurred vision at near? (never, occasionally, often, always); how severe is the blurred vision at near (zero, mild, moderate, severe) and the duration? (3) How often do you experience the itchy eyes, eye swelling and other discomforts (never, occasionally, often, always); how severe is the itchy eyes, eye swelling, other discomforts (zero, mild, moderate, severe) and the duration?

Eye drops and OK lenses

The 0.01% atropine eye drops (pH=5.4-5.6, 3 mL sealed bottle, 15-25°C room temperature storage, discarded eye drops after opening the bottle for 1 month) were prepared by diluting atropine sulfate powder (Shaoxing Minsheng Medical Co., Ltd., Zhejiang, China) with normal saline under sterile conditions and subsequently, adding a preservative (0.3 mg/mL ethyl paraben). The 0.01% atropine eye drops showed minimal degradation (approximately 1.8%) after opening the eye drop bottle for one month, and its properties were stable. A blank solvent without atropine was used as a placebo eye drops. All children were instructed to use 0.01% atropine or placebo eye drops by instilling one drop in both eyes once nightly 10 min before OK lens insertion.
